# Male Accessory Gland Infection: Relevance of Serum Total Testosterone Levels

**DOI:** 10.1155/2014/915752

**Published:** 2014-09-08

**Authors:** R. A. Condorelli, A. E. Calogero, E. Vicari, V. Favilla, S. Cimino, G. I. Russo, G. Morgia, S. La Vignera

**Affiliations:** ^1^Section of Andrology, Endocrinology and Internal Medicine, Department of Medical and Pediatric Sciences, University of Catania, Catania, Italy; ^2^Department of Urology, University of Catania, Catania, Italy

## Abstract

Aim of the present study was to evaluate the different ultrasound characterization of fertile symptomatic patients with MAGI (male accessory gland infection) according to different serum concentrations of total T (TT). We analyzed the ultrasound and hormonal data of 200 patients aged between 24.0 and 67.0 years. Patients were divided into six groups according to the sextile distribution of TT. Patients with serum concentrations of TT < 3.6 ng mL^−1^ had a higher mean duration of symptoms compared to the other examined groups. Patients with serum concentrations of TT > 6.6 ng mL^−1^ showed a frequency of ultrasound criteria suggestive for bilateral form of prostatitis and prostate-vesiculo-epididymitis and significantly lower compared to the other examined groups. At multivariate logistic regression analysis adjusted for age and BMI, TT was an independent predictive factor of prostatovesiculitis (OR = 0.818 [95% CI: 0.675–0.992]; *P* < 0.01) and prostate-vesiculo-epididymitis (OR = 0.714 [95% CI: 0.578–0.880]; *P* < 0.01), which represent the main forms of complicated MAGI. The results of this study suggest that male hypogonadism could be associated with a different ultrasound characterization of these patients.

## 1. Introduction

Male accessory gland infection (MAGI) represents a potential cause of male infertility [1, 2], with a different prevalence in the previous published studies [3].

MAGI may affect sperm parameters through three possible mechanisms: (a) secretory dysfunction of the accessory glands (epididymis, prostate, and seminal vesicles) [4–6], (b) favoring an inflammatory microenvironment (oxygen-free radicals, cytokines) [7–12], and (c) anatomical obstruction of the seminal tract [13, 14].

The ultrasound examination of the epididymis and prostate-vesicular tract represents an important diagnostic tool for the clinical evaluation [9, 15], although it is not considered an essential step in the work-up. Recently, a very comprehensive review on ultrasound physiopathology of male seminal tract has been published [16], confirming the importance of this diagnostic tool for the diagnosis of inflammatory diseases of the male genital tract.

The ultrasound evaluation allows discriminating different diagnostic categories, in particular,

- *uncomplicated form* (ultrasound criteria limited to the prostate: prostatitis (P)),
- *complicated form* (ultrasound criteria extended to seminal vesicles: prostatitis and vesiculitis (PV), and the epididymis: prostatitis, vesiculitis, and epididymitis (PVE)) [9, 15],
- *unilateral form* (unilateral P-PV-PVE) [9, 15, 17],
- *bilateral form* (bilateral P-PV-PVE ) [9, 15, 17]: we have recently reported additional ultrasound criteria that allow the identification of two original ultrasound categories of MAGI [18],
- *hypertrophic-congestive form* (FSUF),
- *fibrosclerotic form* (HCUF).

There are several risk factors that may favor the onset of MAGI, for example, lifestyle, diet, cigarette smoking, gastrointestinal diseases, and sexual promiscuity [19, 20].

The role of low levels of testosterone (T) in promoting the onset of P in an experimental model was recently reported [21]. In particular, a modulatory action of T on inflammatory response in the accessory glands has been demonstrated [22, 23]. However, there is not evidence in the literature regarding the clinical characterization of hypogonadal patients with MAGI.

On the basis of these premises, the aim of this study was to evaluate the different ultrasound characterization of fertile symptomatic patients with MAGI according to different serum concentrations of total T (TT).

## 2. Materials and Methods

Out of a consecutive series of 2000 subjects, we selected 200 fertile (spontaneous pregnancy during the previous 12 months) symptomatic patients with MAGI evaluated during the period between January 2012 and January 2014 that were subjected to evaluation of serum concentrations of TT.

The diagnosis of MAGI was carried out according to the criteria reported in Table 1 and published in several previous studies of our group [1–3, 15]. The diagnosis was further confirmed by the administration of a questionnaire dedicated to patients with MAGI, for the assessment of the severity of the associated symptoms, previously published by our group [24]. This questionnaire (SI-MAGI: structured interview about MAGI) is divided into 4 domains relative to urinary disorders, spontaneous and/or ejaculatory pain and/or discomfort, sexual disorders, and quality of life.

All examined patients had ultrasound criteria suggestive of MAGI, as previously reported [15, 17, 18].  Table 2 shows the ultrasound criteria used by our group and published in previous studies for the confirmation and greater characterization of the clinical diagnosis of MAGI [15, 17, 18].

Subjects with TT higher than the upper limit of the range were excluded. Other exclusion criteria were represented by hormone therapy with antiestrogens or gonadotropins or T.

### 2.1. Hormone Measurements

Hormonal evaluations were performed by electrochemiluminescence with Hitachi-Roche equipment (Cobas 6000, Roche Diagnostics, Indianapolis, IN, USA). The reference intervals were as follows: LH = 1.6–9.0 mIU mL^−1^, FSH = 2.0–12.0 mIU mL^−1^, and total testosterone = 2.8–8.0 ng mL^−1^. Blood sampling was performed at 8:00 am, after at least 8 hours of sleep. The determination of serum LH was repeated after an interval of 30 minutes.

We analyzed the ultrasound and hormonal data (serum TT) of 200 patients with MAGI aged between 24.0 and 67.0 years (mean age = 36.0 ± 14.0 years) with BMI between 18.0 and 33.0 kg/m^2^ (mean value = 25.0 ± 7.0 kg/m^2^).

Patients were divided into six groups according to the sextile distribution of total testosterone:

- *Group 1:* TT ≤ 2.7 ng mL^−1^,
- *Group 2:* TT > 2.7 and ≤3.6 ng mL^−1^,
- *Group 3:* TT > 3.6 and ≤4.4 ng mL^−1^,
- *Group 4:* TT > 4.4 and ≤5.3 ng mL^−1^,
- *Group 5:* TT > 5.3 and ≤6.6 ng mL^−1^,
- *Group 6:* TT > 6.6 ng mL^−1^.

The frequency of the following possible ultrasound conditions was evaluated for each group: P (prostatitis alone), PV (ultrasound criteria suggestive for prostatitis associated with criteria of vesiculitis), and PVE (criteria of prostatitis and vesiculitis associated with epididymitis),* unilateral* P, PV, and PVE,* bilateral* P, PV, and PVE, and HCUF or FSUF.

The study was approved by the Internal Institutional Board and all examined patients signed informed consent to the processing of personal data.

### 2.2. Statistical Analysis

Normality of variables' distribution was tested by Kolmogorov-Smirnov test. Accordingly, variables are presented as means ± standard deviations or median (interquartile range) and differences between groups were tested by Student's independent *t*-test or Mann-Whitney *U* test on the basis of their normal or not-normal distribution, respectively. Pearson's or Spearman's correlation coefficients were used in order to test the associations between the different variables. Multivariate linear regression models adjusted for age and BMI were performed for factors significantly correlated at Spearman's analysis. Multivariate logistic regression analysis was performed to assess significant predictors of MAGI after adjusting for age and BMI.

The software SPSS 9.0 for Windows was used for statistical evaluation (SPSS Inc., Chicago, IL, USA). A statistically significant difference was accepted when the *P* value was lower than 0.05.

## 3. Results

The linear regression analysis showed inverse association between TT and duration of symptoms after adjusting for age and BMI (*r* = −0.929; *P* < 0.01).

At multivariate logistic regression analysis adjusted for age and BMI, TT was an independent predictive factor of P (OR = 1.533 [95% CI: 1.272–1.848]; *P* < 0.01), PV (OR = 0.818 [95% CI: 0.675–0.992]; *P* < 0.01), PVE (OR = 0.714 [95% CI: 0.578–0.880]; *P* < 0.01), UP (OR = 1.847 [95% CI: 1.498–2.278]; *P* < 0.01), BP (OR = 0.615 [95% CI: 0.493–0.766]; *P* < 0.01), BPV (OR = 0.505 [95% CI: 0.321–0.796]; *P* < 0.01), BPVE (OR = 0.610 [95% CI: 0.458–0.812]; *P* < 0.01), HCUF (OR = 2.201 [95% CI: 1.702–2.848]; *P* < 0.01), and FSUF (OR = 0.607 [95% CI: 0.494–0.746]; *P* < 0.01).

Table 3 shows the differences between groups divided into sextiles according to different serum concentration of TT.

## 4. Discussion

The results of this study suggest that different serum concentrations of TT in patients with MAGI are associated with a different ultrasonographic characterization.

In particular, the reduction of TT represents a predictive factor for ultrasonographic complicated form (PV and PVE), ultrasonographic bilateral form of P, PV, and PVE, and ultrasonographic fibrosclerotic form. The increase in TT represents a predictive factor for ultrasonographic uncomplicated form (P), ultrasonographic unilateral form of P, and ultrasonographic hypertrophic-congestive form.

The comparison between groups showed a significantly lower frequency of PVE (ultrasonographic form with greater anatomic extension) in group 6 (TT > 6.6 ng mL^−1^). The same group showed a significantly higher frequency of the ultrasonographic hypertrophic-congestive form and a significantly lower frequency of the ultrasonographic fibrosclerotic form.

Finally, patients with TT ≤ 3.6 ng mL^−1^ showed a significantly greater duration of symptoms of MAGI compared to the other groups.

Therefore, it is conceivable that, in clinical practice, the male hypogonadism could represent a risk factor for a complicated form of MAGI.

There are no other studies in the literature that have examined the frequency and/or the clinical characteristics of patients with MAGI according to the different levels of TT; however, it is known that MAGI represent a very important clinical problem for the symptoms and the reproductive consequences [3, 24, 25].

The low levels of TT could have an important significance from the diagnostic and therapeutic point of view. In fact, the results of this study suggest that male hypogonadism could be a risk factor for the development of MAGI and in particular for complicated form of MAGI (ultrasound criteria suggestive for extension to the seminal vesicles and epididymis). Furthermore, the hypogonadal patients showed a higher frequency of “bilateral” and “fibrosclerotic” ultrasound form of MAGI.

Ultrasound evaluation of the epididymis, prostate, and seminal vesicles represents an important tool for the clinical characterization of MAGI; however, it is not a mandatory diagnostic step [3]. In this field, the experience of our group showed that the progressive involvement of more accessory glands detectable by the ultrasound evaluation is associated with a poor quality of sperm parameters [3] and severe symptoms [24, 25]. Moreover, the ultrasound examination allows discriminating the bilateral form of MAGI better than some semen parameters such as the fructose [17]. We recently reported two other variants of MAGI, respectively, called “hypertrophic-congestive” and “fibrosclerotic” ultrasound form, associated with a different sperm quality after pharmacological treatment (lower in patients with “fibrosclerotic” form) [18]. Finally, the ultrasound evaluation allows discriminating the patients with persistent bacteriospermia [26]. Even more recently, two other categories of patients (diabetes mellitus [27] and irritable bowel syndrome [20]) have been characterized by ultrasound examination for the peculiar characteristics of the sex accessory glands.

The evaluation of serum levels of TT is often required during an initial andrological consultation for various clinical problems (sexual or reproductive pathology) and therefore we wanted to examine retrospectively the frequency of ultrasound criteria suggestive of MAGI according to the different serum concentrations of TT. From the methodological point of view, there are no similar studies in the literature; however, for the interpretation of the results, it is necessary to analyze some evidences that suggest a role of testosterone as a modulator of the inflammatory response in the prostatic tissue.

Several mechanisms have been proposed to explain the potential anti-inflammatory effects of T on the prostatic tissue. In particular, it has been suggested that T could reduce the tissue expression of proinflammatory cytokines and T-lymphocytes and antagonize macrophage activation and neutrophil in the prostatic tissue and the progression of the fibrosis. Furthermore, T seems to positively modulate the expression of junctional proteins of the prostatic epithelium and negatively the activation of the immune response. Finally, the antiestrogenic effect and the negative modulation of the expression of toll-like receptor 4 represent other mechanisms [21–23, 28–30].

In detail, the study of Vignozzi and colleagues [21] showed that T reduced the expression of inflammatory prostatic markers in an experimental model of prostatic inflammation induced in rabbits by experiment fed with high-calorie diet, modulating the expression of IL8, IL6, IL1*β*, and TNF (proinflammatory cytokines), T lymphocytes, macrophages, markers of neutrophil activation, and prostatic fibrosis. The hypogonadism is associated with a reduced expression of Claudina 4 and Claudina 8, the essential components in the formation of junctional structures of the prostatic epithelium, whose alteration induces inflammatory degeneration of the prostate tissue and the immune response [22]; instead, the T supplementation is associated with reexpression of these proteins [22]. A reduction of T/estradiol ratio is associated with a higher frequency of prostatic inflammation in an experimental model [28]. Finally, in castrated rats, the expression of toll-like receptor 4, functional complex involved in the activation of inflammatory and autoimmune response, is significantly increased [29].

Finally, the patients of group 1 have a condition of male hypogonadism probably related to the age, suggested by higher serum concentrations of LH and lower mean value of testicular volume. This aspect must be evaluated through further studies in order to understand a possible association between persistence of MAGI (these patients have an increased duration of symptoms) and the different serum concentrations of TT, as suggested in previous studies about a similar clinical model represented by LUTS (lower urinary tract symptoms) secondary to benign prostatic hyperplasia (BPH) [31, 32].

An important difference between these two clinical models is represented by the increase in prostate volume for the patients with LUTS-BPH related that represent a potential contraindication to the hormonal treatment with T, while patients with MAGI have an inflammation of periurethral zone assessed by ultrasound evaluation associated with obstructive symptoms [11, 15, 17, 18], without a real increase in prostate volume. These patients may find advantages from hormone therapy with T when hypogonadism is associated; however, further studies will need to evaluate this aspect. However, it should be noted that increasing evidence shows that prostate volume and activity are T dependent only in the overt hypogonadal range, for very low serum T concentrations [33, 34].

In conclusion, the results of this study suggest evaluating the serum concentrations of total testosterone in patients with MAGI. In particular, male hypogonadism is associated with different clinical and ultrasound characterization of these patients.

## Figures and Tables

**Table 1:** Clinical criteria for diagnosis of MAGI.

(a) History of urogenital infection and/or abnormal rectal palpation.	

(b) Significant alterations in the expressed prostatic fluid and/or urinary sediment after prostatic massage.	

(c) (1) Uniform growth of more than 10(3) pathogenic bacteria, or more than 10(4) nonpathogenic bacteria per mL, in culture of diluted seminal plasma. (c) (2) Presence of more than 10(6) (peroxidase positive) leucocytes per mL of ejaculate. (c) (3) Signs of disturbed secretory function of the prostate or seminal vesicles.	

Diagnosis is accepted if at least 2 criteria are present: (i) a + b, (ii) a + c (1 or 2 or 3), (iii) b + c (1 or 2 or 3), (iv) c1 + c2, (v) c1 + c3, (vi) c2 + c3.	

**Table 2:** Ultrasound criteria for the confirmation and greater characterization of the clinical diagnosis of MAGI.

**Prostatitis is suspected in the presence of >2 of the following ultrasonographic signs: **	
(1) asymmetry of the gland volume, (2) areas of low echogenicity, (3) areas of high echogenicity, (4) dilatation of periprostatic venous plexus, (5) single or multiple internal similar cystic areas, and (6) area/s of moderate increase in vascularity (focal or multiple).	

**Vesiculitis is suspected in the presence of >2 of the following ultrasonographic signs:**	
(1) increased (>14 mm) anteroposterior diameter mono- or bilateral, (2) asymmetry >2.5 mm (normal 7–14 mm) compared to the contralateral vesicle, (3) reduced (<7 mm) anteroposterior diameter mono- or bilateral, (4) glandular epithelium thickened and/or calcified, (5) polycyclic areas separated by hyperechoic septa in one or both vesicles, (6) fundus/body ratio >2.5, (7) fundus/body ratio <1, and (8) anteroposterior diameter unchanged after recent immediate ejaculation.	

**Epididymitis is suspected in the presence of >2 of the following ultrasonographic signs:**	
(1) increase in size of the head (craniocaudal diameter >12 mm) and/or of the tail (craniocaudal diameter > 6 mm) (finding single or bilateral), (2) presence of multiple microcystis in the head and/or tail (finding single or bilateral), (3) low echogenicity or high echogenicity mono- or bilateral, (4) large hydrocele mono- or bilateral, (5) enlargement in superior part of the cephalic tract and superior/inferior part ratio >1, and (6) unchanged anteroposterior diameter of tail after ejaculation.	

**Table 3:** Differences between groups divided into sextiles according to the values of TT.

	Group 1 (TT ≤ 2.7) (*n* = 34)	Group 2 (TT > 2.7 and ≤3.6) (*n* = 33)	Group 3 (TT > 3.6 and ≤4.4) (*n* = 37)	Group 4 (TT > 4.4 and ≤5.3) (*n* = 30)	Group 5 (TT > 5.3 and ≤6.6) (*n* = 36)	Group 6 (TT > 6.6) (*n* = 30)
Age, median (IQR)	40.0 (36.0–42.0)	41.0 (33.5–42.0)	38.0 (31.5–42.0)	33.0 (30.0–40.5)∗	36.0 (28.0–40.0)∗	30.0 (26.0–39.0)∗
BMI, median (IQR)	26.5 (23.0–30.25)	26.0 (23.0–30.0)	24.0 (23.0–28.0)	24.5 (23.0–28.0)	23.0 (23.0–25.75)∗	22.0 (22.0–23.5)∗
FSH, median (IQR)	3.2 (3.0–3.42)	3.0 (2.85–3.2)	3.0 (2.8–3.1)	3.05 (2.8–3.2)	2.8 (2.35–3.0)∗	2.5 (2.2–2.9)∗
LH, median (IQR)	6.5 (6.0–7.2)	6.0 (4.7–6.1)	4.5 (4.2–5.1)∗	4.2 (4.07–4.6)∗	4.2 (3.52–4.6)∗	3.8 (3.1–4.2)∗
Duration of symptoms, median (IQR)	28.0 (15.0–21.0)	16.0 (15.0–28.0)	9.0 (7.0–12.0)∗	6.0 (5.0–8.0)∗	5.0 (4.0–6.0)∗	3.0 (2.0–4.0)∗
Testicular volume, median (IQR)	8.0 (6.75–10.0)	10.0 (8.5–12.0)∗	15.0 (14.0–17.0)∗	17.0 (15.0–18.0)∗	19.0 (17.2–21.0)∗	22.0 (20.0–23.0)∗
Microbial forms (%)	4 (11.8)	5 (15.2)	8 (21.6)	4 (13.3)	3 (8.3)	4 (13.3)
P	7 (7.9)	9 (10.1)	16 (18.0)	16 (18.0)	22 (24.7)	19 (21.3)^†^
PV	12 (21.1)	12 (21.1)	10 (17.5)	9 (15.8)	9 (15.8)	5 (8.8)
PVE	14 (26.9)	12 (23.1)	8 (15.4)	8 (15.4)	7 (13.5)	3 (5.8)^†^
UP	5 (6.5)	3 (3.9)	13 (16.9)	16 (20.8)	20 (26.0)	20 (26.0)^†^
BP	31 (27.0)	27 (23.5)	19 (26.5)	14 (12.2)	17 (14.8)	7 (6.1)^†^
UPV	7 (17.5)	7 (17.5)	8 (20.0)	8 (20.0)	7 (17.5)	3 (7.5)
BPV	6 (35.3)	5 (29.4)	2 (11.8)	1 (5.9)	2 (11.8)	1 (5.9)
UPVE	4 (16.7)	4 (16.7)	3 (12.5)	5 (20.8)	7 (29.2)	1 (4.2)
BPVE	10 (33.3)	8 (26.7)	5 (16.7)	2 (6.7)	4 (13.3)	1 (3.3)^†^
HCUF	2 (3.0)	3 (4.5)	10 (15.2)	14 (21.2)	19 (18.8)	18 (27.3)^†^
FSUF	22 (30.1)	17 (23.3)	12 (16.4)	9 (12.3)	10 (13.7)	3 (4.1)^†^

IQR: interquartile range; ∗*P* < 0.05 versus Group 1; ^†^ *P* < 0.05 at chi-square test.

UP: unilateral prostatitis; BP: bilateral prostatitis; UPV: unilateral prostatovesiculitis; BPV: bilateral prostatovesiculitis; UPVE: unilateral prostato-vesiculo-epididymitis; BPVE: bilateral prostato-vesiculo-epididymitis.
